# Shape and Rule Information Is Reflected in Different Local Field Potential Frequencies and Different Areas of the Primate Lateral Prefrontal Cortex

**DOI:** 10.3389/fnbeh.2022.750832

**Published:** 2022-05-13

**Authors:** Kazuhiro Sakamoto, Norihiko Kawaguchi, Hajime Mushiake

**Affiliations:** ^1^Department of Neuroscience, Faculty of Medicine, Tohoku Medical and Pharmaceutical University, Sendai, Japan; ^2^Department of Physiology, Tohoku University School of Medicine, Sendai, Japan

**Keywords:** monkey, lateral prefrontal cortex, shape manipulation task, visual object, behavioral rule, ventral-lateral distinction, theta-delta wave, gamma wave

## Abstract

The lateral prefrontal cortex (LFPC) plays a crucial role in executive function by adaptively storing behavior-relevant information as working memory. Neural mechanisms associated with local field potentials (LFPs) may underlie the adaptive properties of the LFPC. Here, we analyzed how LFPs recorded from the monkey LFPC are modulated by the crucial factors of a shape manipulation task. In this task, the test shape is transformed by manipulating a lever to match the size and orientation of the sample shape. The subject is required to temporarily memorize the rules such as the arm-movement-manipulation relationship and the sample shape to generate the sequential behavior of operations. In the present study, we focused on task variables about shape and rules, and examined among which aspects distinguish the ventral and dorsal sides of the LFPC. We found that the transformed shape in the sample period strongly affected the theta and delta waves in the delay period on the ventral side, while the arm-manipulation assignment influenced the gamma components on the dorsal side. These findings suggest that area- and frequency-selective LFP modulations are involved in dynamically recruiting different behavior-relevant information in the LFPC.

## Introduction

The lateral prefrontal cortex (LFPC) plays a crucial role in executive function, i.e., problem solving and action planning in various environments (Duncan, 2001; Saito et al., 2005; Mushiake et al., 2006; Tanji et al., 2007; Sakamoto et al., 2008, 2013, 2020b; Tanji and Hoshi, 2008; Katori et al., 2011; Passingham and Wise, 2012; Fuster, 2015). The LFPC exists at a nodal point of the hierarchical structure from perception/recognition to behavior/movement (Felleman and Van Essen, 1991), where information from percepts about the external world to internal behavioral norms is integrated (e.g., Petrides et al., 2012) and temporarily stored as working memory (Fuster and Alexander, 1971; Fuster, 1973; Kojima and Goldman-Rakic, 1982, 1984; Funahashi et al., 1989; Wilson et al., 1993; Miller et al., 1996; Rao et al., 1997; Rainer et al., 1998). The coding of information in the LFPC is expected to be flexible depending on the environmental demand (Duncan, 2001), although the neural mechanism underlying flexible recruitment of behaviorally relevant information has not yet been fully elucidated.

Local field potentials (LFPs) are potentials with a relatively low frequency component that are recorded extracellularly in the brain. In recent years, they have received much attention for their roles in flexible and context-dependent information transmission in the brain (Akam and Kullmann, 2014). Such flexibility is also thought to be associated with adaptive information processing in the LFPC. Indeed, specific frequency changes dependent on task event and task content have been reported in the LFPC of macaque monkeys (Buschman et al., 2012; Sakamoto et al., 2015, 2020a; Lundqvist et al., 2016; Wimmer et al., 2016; Ma et al., 2018; Wutz et al., 2018; Dezfouli et al., 2021). All of these suggest that different frequency components make different contributions to different aspects of LFPC function.

The LFPC is anatomically distinguished into ventral and dorsal sides. The ventral side has connections with the orbitofrontal cortex and the temporal lobe known to be involved in object recognition, while the dorsal side has mutual interactions with the parietal lobe involved in spatial perception and the medial frontal lobe involved in internal states (Petrides and Pandya, 1994; Averbeck and Seo, 2008; Yetarian et al., 2012). Consistent with these anatomical backgrounds, neuronal activities reflecting visual objects themselves or the information they contain have been reported from the ventral LFPC of monkeys (Wilson et al., 1993; Ninokura et al., 2004), while the dorsal region is involved in the retrieval of task-related information and its manipulation for action planning and execution (Hoshi and Tanji, 2004; Ninokura et al., 2004). Although there have been reports on LFPs in the LFPC, there are only a few studies of LFPs that distinguished between the ventral and dorsal sides (Wutz et al., 2018; Sakamoto et al., 2020a). It remains to be investigated how the functional differentiation of the LFPC corresponds to the dynamic properties of LFPs.

**FIGURE 1:**
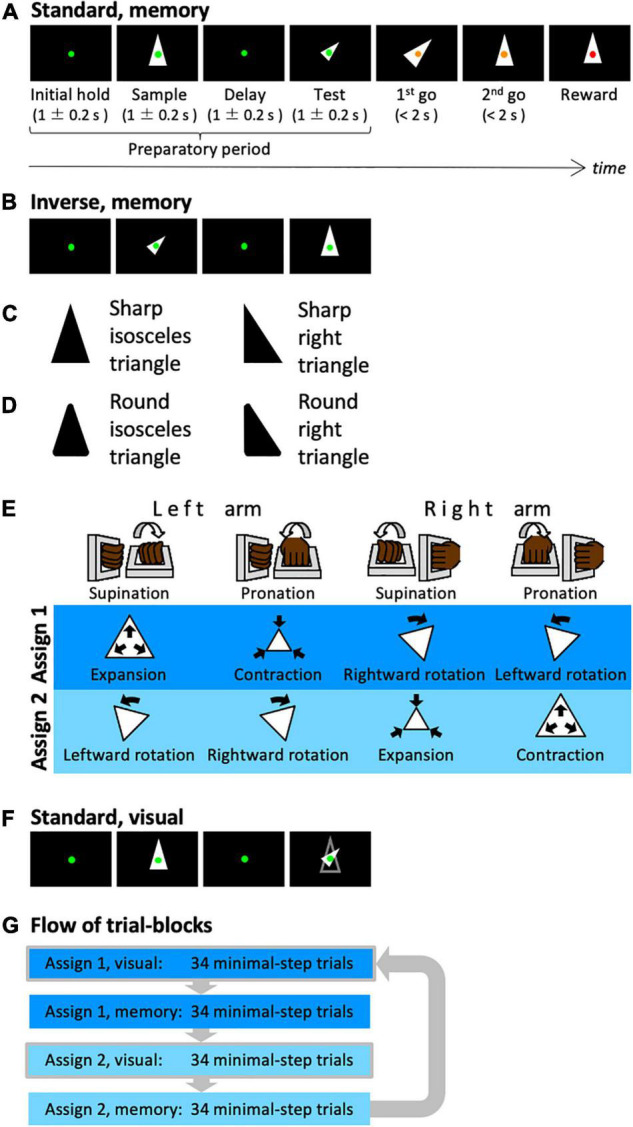
Shape manipulation task. **(A)** The time-course of one trial. In this case, a shape with standard size and upward orientation was presented in the sample period, while the transformed shape was presented in the test period (standard version). **(B)** In this case, the transformed shape was presented in the sample period. **(C)** The types of the presented shapes. An isosceles or right triangle was used. **(D)** The local features of the presented shapes. Sharp or round corners were used. **(E)** Assignments between arm movements and manipulation of the shape. **(F)** In this case, the contour of the sample shape was presented in the test period (visual). The subject can recognize the transformation between the sample cue and the test cue visually, i.e., without memory. **(G)** Illustration of the composition of the trial blocks.

Here, we analyzed how LFPs recorded from the monkey LFPC are modulated by the important factors of a shape manipulation task. In this task, the test shape is transformed by manipulating a lever to match the size and orientation of the sample shape (Figure 1A; Sakamoto et al., 2015, 2020a). The subject is required to temporarily memorize the rules, such as the arm-movement-manipulation relationship and the sample shape, to generate the sequential behavior of the operations. In a previous study that analyzed the overall time-frequency trend, we found that the ventral and dorsal sides could be distinguished based on theta and gamma wave features (Sakamoto et al., 2020a). Hence, in the present study, we focused on these frequency ranges and memories of shape and rules, and examined their changes in different time periods of the task to functionally distinguish the ventral and dorsal sides.

## Materials and Methods

### Subjects

Two male Japanese monkeys (*Macaca fuscata*) weighing 9.0 kg (Monkey 1) and 8.5 kg (Monkey 2) were used in the present experiment. These monkeys had previously participated in published studies (Sakamoto et al., 2015, 2020a). All experimental protocols were approved by the Animal Care and Use Committee of Tohoku University (Permit # 20MeA-2), and all animal protocols conformed with the National Institutes of Health Guidelines for the Care and Use of Laboratory Animals, as well as with the recommendations of the Weatherall Report.

### Behavioral Task

The monkeys were trained to perform a shape manipulation task (Figure 1A). The goal was to fit the test shape to the sample shape during a trial. First, a green fixation spot appeared on the screen (initial hold period) before a sample shape was displayed for 1 ± 0.2 s (sample period). Then, after a 1 ± 0.2 s interval (delay period), a test shape that was homothetic to the sample shape but transformed (i.e., expanded/contracted and rotated) was displayed for 1 ± 0.2 s (test period). Subsequently, the color of the fixation point was changed to yellow; this served as a go signal to initiate the first-step movement (first go). Then the monkey was required to execute a movement within the time window of 2 s and wait until the second go signal (second go) appeared. When the monkey completed one movement, the fixation point returned to green and a change in color to yellow was used as a go signal again. At each go signal, the monkeys were allowed to make a single one-arm movement and were also permitted to perform any number of steps if a movement was executed within the time window. When the test shape was successfully transformed to fit the sample shape, the fixation point turned red and the monkeys were rewarded with a drop of an isotonic drink. Each trial was set to require at least two steps to be rewarded.

There are two types of trials: standard trials, in which a shape of standard size and orientation is presented during the sample period, and inverse trials, in which the standard shape is presented during the test period (Figure 1B). By presenting the standard and inverse trials in the same proportion randomly, the animals were forced to plan their sequential behavior by comparing the shapes of the sample and test periods. Thereby, the animals had to remember the shape of the sample period. In addition to these two types, trials in which the same arm was used for the first and second steps were conducted (same arm twice trials). For instance, two rotation operations using the same arm were required in the trial where the shapes in the sample and test phases were oriented 90 degrees to one another. By mixing the same arm twice trials from time to time, it is possible to avoid the situation in which the arm to be used for the second step was automatically decided once the first step was decided.

In each trial, a single shape was randomly selected from a set of shapes. We used two types of shapes, isosceles and right triangles (Figure 1C), and two types of local features, sharp and rounded corners (Figure 1D); i.e., four shape-specific attributes.

The shape manipulations were linked to the movements of two manipulanda installed on the chair and operated with the right or left wrist. To dissociate movements of the arms from manipulative operations of the test shape, the monkeys were trained to perform the task with two different arm-manipulation assignments (Figure 1E). For the first cursor assignment (assign 1), left-arm supination and pronation controlled expansion (double the area) and contraction (half the area), respectively, of the test shape and right-arm supination and pronation controlled rightward (–45°) and leftward rotation (45°), respectively, of the test shape. For the second cursor assignment (assign 2), left-arm supination, left-arm pronation, right-arm supination, and right-arm pronation were assigned to leftward rotation, rightward rotation, contraction, and expansion, respectively.

In this task, we used a correction method. The task conditions of incorrect or non-minimal step trials were repeated until the correct answer was obtained in the minimal step trial (two steps). The arm-movement assignment described above was switched for each of the 68 minimal step trials. In the first half of each 68-trial block (34 trials), the contour of the sample cue was displayed during the test cue period (visually guided task: Figure 1F), whereas the contour was not displayed in the latter half (memory-guided task, 34 trials). Each block of 34 trials contained 16 standard and inverse trials, and 2 same arm twice trials. Within a block, each condition was presented pseudo-randomly. These trial blocks were executed in the order shown in Figure 1G.

### Electrophysiological Recordings

**FIGURE 2:**
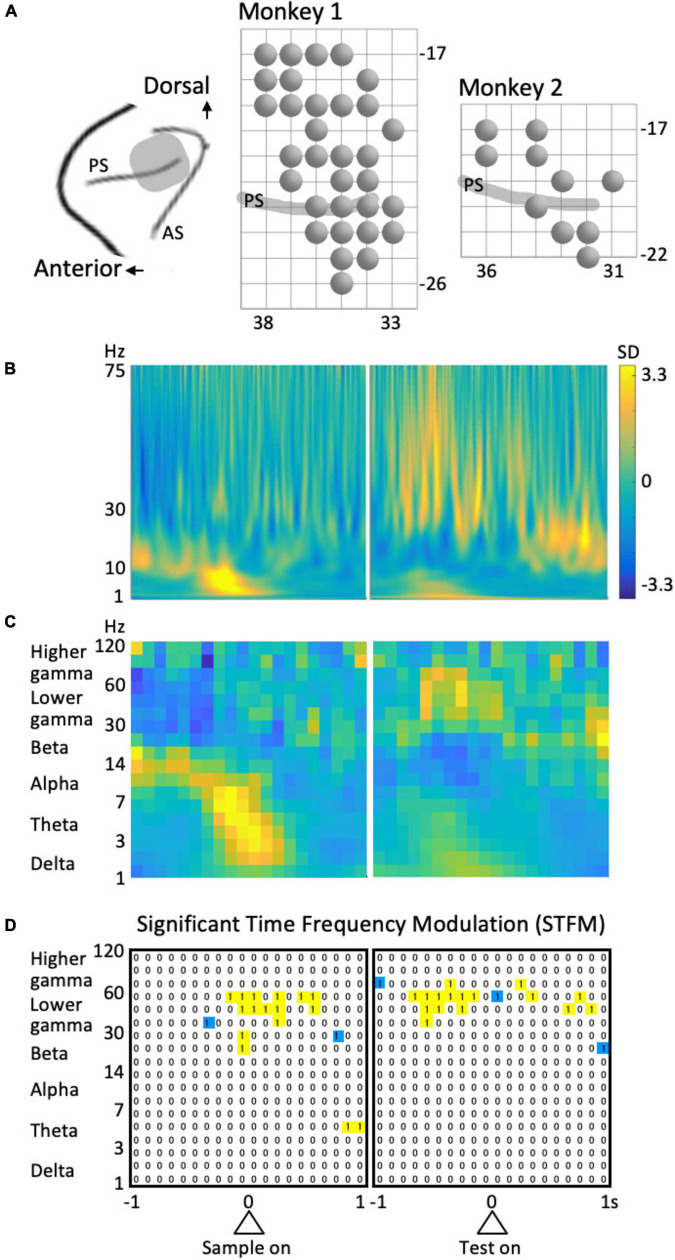
Recording and analysis of LFPs. **(A)** Recording sites. PS, principal sulcus; AS, arcuate sulcus. A circle represents a recording site. The grid interval is 1 mm. **(B)** Example of the time-frequency spectrum averaged across all trials. Same site as in Figure 3 (left column). **(C)** The coarse-grained time-frequency spectrum obtained from **(B)**. **(D)** An example of the results of stepwise regression analysis for the data shown in Figure 4 (left column). “1” Represent the time-frequency region that provided a significant model including the predictor variable of interest. To avoid multi-comparison problems, isolated regions (blue) were excluded.

The surgical procedure used in the present study has previously been described (Sakamoto et al., 2008, 2015). Following surgery, cortical sulci were identified using a magnetic resonance imaging scanner (OPART 3D-System; Toshiba, Tokyo, Japan) and by mapping single-unit activities that were recorded using conventional metal electrodes. The recording areas in this study is involved in are in the left hemisphere as indicated in Figure 2A.

**FIGURE 3:**
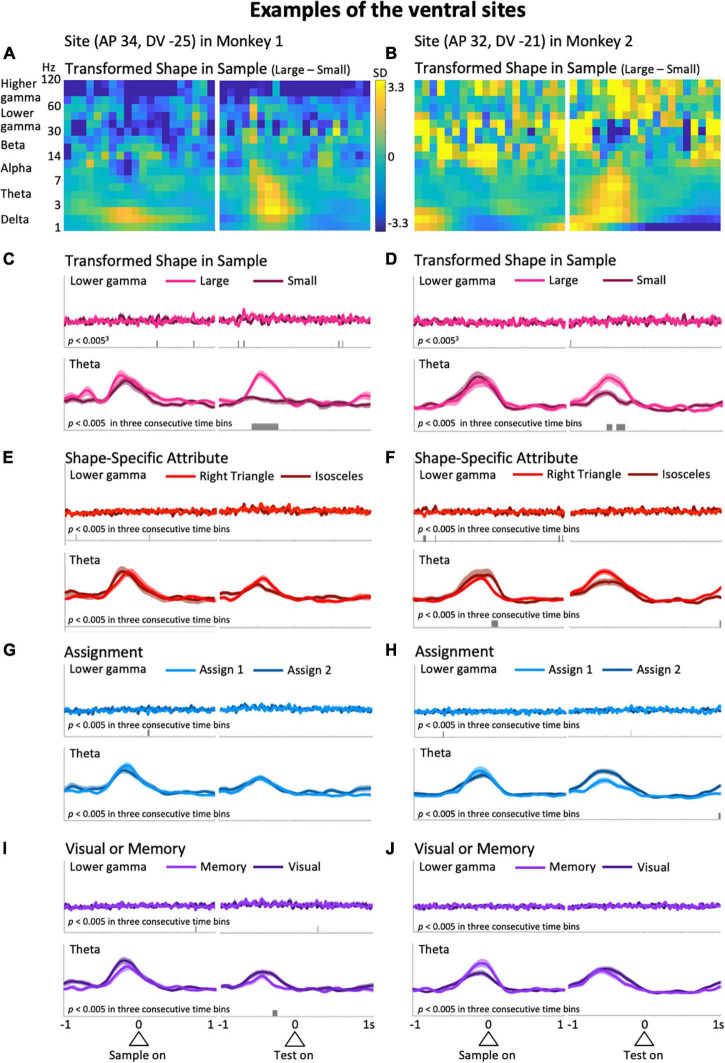
Typical examples from the ventral side of the LFPC in the two monkeys. The left column was obtained from the site with coordinates (AP 34, DV –25); i.e., 34 and –25 mm in the anterior-posterior and dorsal-ventral axes, respectively, while the right column is from (AP 32, DV –21). **(A,B)** The difference in the time-frequency spectra between large and small sizes of the shape presented in the sample period. **(C,D)** The mean upper envelopes of lower-gamma (top) and theta (bottom) waves sorted based on the sample-shape scale factor. **(E–J)** The mean upper envelopes sorted based on variables of shape-specific attributes **(E,F)**, assignment **(G,H)**, and visual or memory **(I,J)**. The scales for the mean upper envelopes were identical in each column. Small bars in each mean upper envelope plot indicate the time regions showing significant differences (*p* < 0.005) in three consecutive time bins.

**FIGURE 4:**
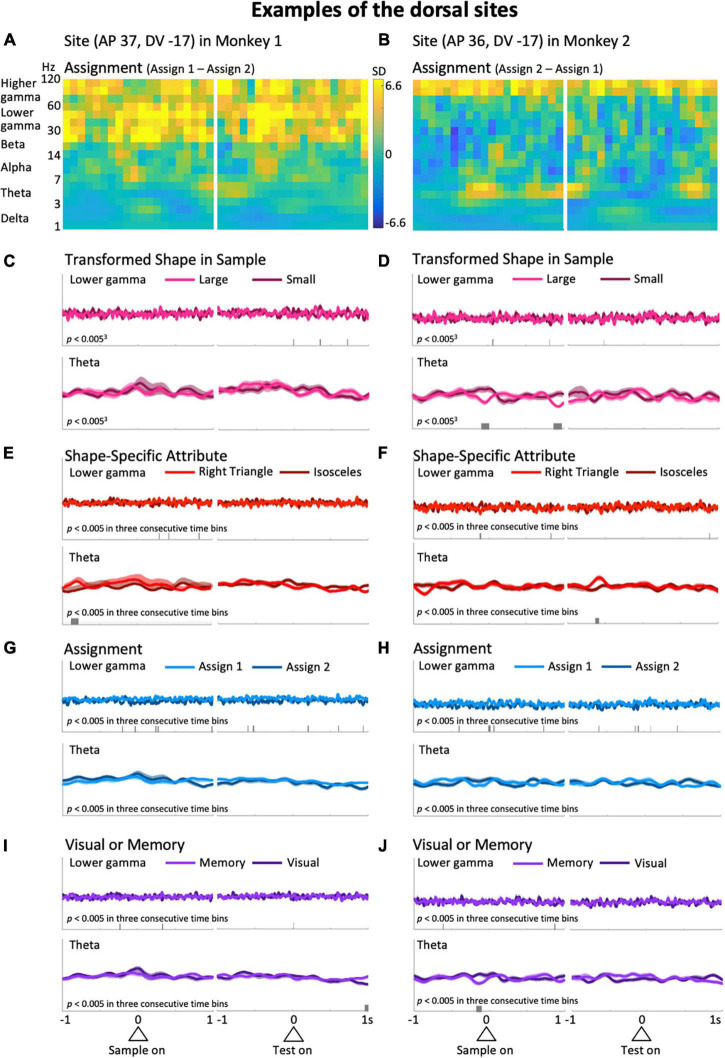
Typical examples from the dorsal side of the LFPC in Monkeys 1 (left column: AP 37, DV -17) and 2 (right column: AP 36, DV -17). **(A,B)** Differences of the time-frequency spectra between assign 1 and assign 2. **(C–J)** Same as the corresponding plots in Figure 3. The scales for the mean upper envelopes are identical in each column. Small bars in each mean upper envelope plot indicate the time regions of significant differences (*p* < 0.005) in three consecutive time bins.

All electrophysiological recordings were performed using linear array multi-contact electrodes (U-Probe; Plexon Inc., Dallas, TX, United States) that contained 15 recording contacts (impedance: 0.3–1.3 MΩ at 1 kHz) with an inter-contact spacing of 150 or 200 μm. The electrode was penetrated with a fixed angle almost perpendicular to the cortical surface except for the regions near the principal sulcus. A guide needle was used to introduce the electrode and once the electrode reached the dura mater, advancement of the guide needle was stopped so the electrode could be inserted into the cortex; each electrode was precisely positioned. The electrodes were lowered until the multi-unit activity that was initially encountered through the bottommost contact (ch. 15) was detected through the top contact (ch. 1). Signals from the electrode were collected using a data acquisition system (Neuralynx, Bozeman, MT, United States) and LFPs and spikes were obtained by band-pass filtering the raw signal from 0.1 to 475 Hz and from 600 Hz to 6 kHz, respectively.

In the following analyses, LFPs recorded from the bottommost contact were used because of the small variability of the spectral patterns compared to those from the upper layers.

### Data Analysis

The data from all trials were included in the present analyses. LFPs during the 4 s of the preparatory period were transformed into small time-frequency regions of 0.05-octave and 1/1,240 s using Morlet wavelets with the center frequencies of the kernels ranging from 1 to 256 Hz. The percentages of energy for each transform coefficient were obtained at each moment and averaged across trials to obtain a time-frequency LFP spectrogram for each recording site. In addition, an overall LFP spectrum was obtained by averaging across time and subtracting from the spectrum at each time of the LFP spectrogram. The values were normalized to the standard deviation (SD) at each frequency to yield a normalized LFP spectrogram (Figure 2B).

The normalized LFP spectrum was coarse-grained into 100 ms to 0.35 octave time-frequency domains (Figure 2C) and divided into six frequency ranges: delta (1–3 Hz), theta (3–7 Hz), alpha (7–14 Hz), beta (14–30 Hz), lower gamma (30–60 Hz), and higher gamma (60–120 Hz). The time intervals for analysis were 1 s before and after the sample stimulus onset and 1 s before and after the test stimulus onset; i.e., 4 s of the preparatory period (see Figure 1A). Therefore, the data from each frequency included 3 × 40 time-frequency domains (Figure 2C).

We executed a stepwise linear-regression analysis of the LFP data for each explanatory factor or predictor variable. The analysis was done for each time-frequency domain mentioned above. We used the “stepwiselm” function of MATLAB, and started from “constant,” using the “see” criterion. That is, the criterion to add or remove predictor variables was *p*-value for an *F*-test of the change in the sum of squared error by adding or removing the term. We used these options because these provided the strictest results. The *p*-values used were the defaults: 0.05 for addition and 0.10 for removal. We excluded interaction terms from the analysis for simplicity.

The predictor variables in the stepwise linear regression analysis included the following. For transformed shapes, there were four variables: size of the shape in the sample period (Transformed Shape in Sample Scale), rotation of the shape in the sample period (Transformed Shape in Sample Rotation), size of the shape in the test period (Transformed Shape in Test Scale), and rotation of the shape in the test period (Transformed Shape in Test Rotation). As for shape-specific attributes, we considered the type of shape (i.e., Shape-Specific Attributes Isosceles or Right Angle) (Figure 1C) as well as the type of feature (i.e., Shape-Specific Attributes Round or Sharp) (Figure 1D). The two variables related to the rules of the task were the assignment between the arm-movements and the operation of shape manipulation (Assignment: Figure 1E), and the distinction between memory-guided and visually guided (Visual or Memory: Figure 1F). We focus our discussion on these shape- and rule-related variables. The other three variables of animal performance are the distinction between correct and incorrect trials (Correct or Error), the distinction between minimal-step and non-minimal step correct trials (Minimal Steps), and whether the previous trial was an error/non-minimal step trial, i.e., whether the same condition as in the previous trial is repeated (Repeated Trial). For the task structure, there are two variables: whether the standard shape is presented in the sample or test phase (Normal or Inverse, Figure 2F), and whether the same arm is required to be used in both the first and second steps (Same Arm Twice Trial). In addition, we used four variables for each of the operations performed on the first and second steps (Manipulation in Step 1 Expansion or Contraction, Manipulation in Step 1 Leftward or Rightward, Manipulation in Step 2 Expansion or Contraction, and Manipulation in Step 2 Leftward or Rightward). Similarly, there were a total of four variables for arm movements in the two steps, including which arm was used and whether the movement was supination or pronation (Arm Movement in Step 1 Left Arm or Right Arm, Arm Movement in Step 1 Pronation or Supination, Arm Movement in Step 2 Left Arm or Right Arm, and Arm Movement in Step 2 Pronation or Supination). Variables were also used for the presence or absence of action at each step (Action in Step 1, and Action in Step 2). In total, 23 factors were used in the stepwise linear regression analysis. Table 1 lists the correlations among these predictor variables.

**TABLE 1:** Correlations between predictor variables during the recording session of the example shown in Figure 3 left column.

	A	VM	CE	MS	RT	SSAIRA	SSARS	NI	SATT	TSSS	TSSR	TSTS	TSTR	MS1EC	MS1LR	MS2EC	MS2LR	AMS1LR	AMS1PS	AMS2LR	AMS2PS	AS1	AS2
Assignment (A)	1.000	0.056	0.063	0.031	0.003	0.022	–0.034	–0.016	–0.071	0.064	–0.031	0.026	–0.122	–0.430	0.126	0.338	–0.018	–0.055	–0.102	0.047	0.048	–	0.113
Visual or Memory (VM)		1.000	–0.093	–0.077	0.157	–0.028	–0.033	–0.023	–0.126	0.067	0.064	–0.084	–0.107	0.041	–0.090	0.028	0.217	0.046	–0.070	–0.165	0.062	–	–0.100
Correct or Error (CE)			1.000	0.511	0.088	0.010	0.103	–0.027	–0.019	0.101	–0.114	–0.013	0.005	0.075	0.007	0.050	–0.021	–0.141	0.027	0.222	–0.010	–	0.491
Minimal Steps (MS)				1.000	0.172	–0.092	0.033	–0.154	–0.338	0.108	–0.050	0.064	–0.122	0.017	0.042	0.112	0.028	–0.008	0.049	0.241	–0.151	–	0.251
Repeated Trial (RT)					1.000	0.033	–0.072	0.104	0.155	–0.073	0.144	–0.025	0.037	0.025	0.036	–0.042	0.048	0.138	–0.210	–0.120	0.140	–	0.043
Shape-Specific Attribute Isosceles or Right Angle (SSAIRA)						1.000	0.002	0.089	0.122	–0.045	–0.078	–0.086	0.055	0.063	–0.148	–0.039	0.062	0.030	0.057	–0.035	0.064	–	0.005
Shape-Specific Attribute Round or Sharp (SSARS)							1.000	0.040	0.071	0.014	–0.048	–0.107	0.009	0.140	–0.019	–0.024	0.018	0.007	–0.024	0.047	0.125	–	0.105
Normal or Inverse (NI)								1.000	0.293	–0.041	–0.014	–0.017	0.086	–0.014	–0.092	0.032	–0.039	0.077	–0.013	–0.095	0.077	–	0.014
Same Arm Twice Trial (SATT)									1.000	–0.010	–0.177	–0.115	0.180	0.101	–0.111	–0.033	–0.077	–0.055	–0.025	–0.020	0.205	–	0.036
Transformed Shape in Sample Scale (TSSS)										1.000	–0.126	0.008	–0.031	0.309	–0.114	0.367	0.159	–0.417	–0.150	0.329	–0.047	–	0.068
Transformed Shape in Sample Rotation (TSSR)											1.000	0.031	–0.010	0.010	0.364	–0.106	0.334	–0.002	–0.061	–0.047	0.061	–	–0.074
Transformed Shape in Test Scale (TSTS)												1.000	0.012	–0.433	0.089	–0.322	–0.188	0.423	–0.021	–0.346	–0.127	–	0.069
Transformed Shape in Test Rotation (TSTR)													1.000	–0.015	–0.397	–0.013	–0.387	0.060	0.068	–0.072	–0.051	–	–0.072
Manipulation in Step 1 Expansion or Contraction (MS1EC)														1.000	–0.371	–0.199	0.388	–0.331	0.196	0.329	0.314	–	–0.055
Manipulation in Step 1 Leftward or Rightward (MS1LR)															1.000	0.071	–0.246	–0.070	–0.077	0.040	–0.053	–	–0.058
Manipulation in Step 2 Expansion or Contraction (MS2EC)																1.000	–0.273	–0.377	–0.253	0.356	–0.211	–	0.058
Manipulation in Step 2 Leftward or Rightward (MS2LR)																	1.000	–0.078	0.013	0.000	0.160	–	0.057
Arm Movement in Step 1 Left Arm or Right Arm (AMS1LR)																		1.000	–0.038	–0.807	–0.226	–	–0.014
Arm Movement in Step 1 Pronation or Supination (AMS1PS)																			1.000	0.076	0.160	–	0.043
Arm Movement in Step 2 Left Arm or Right Arm (AMS2LR)																				1.000	0.148	–	0.109
Arm Movement in Step 2 Pronation or Supination (AMS2PS)																					1.000	–	0.081
Action in Step 1 (AS1)																						–	–
Action in Step 2 (AS2)																							1.000

To detect the time-frequency domains of significant time-frequency modulation (STFM), we first extracted the domains that provided a model with a significance level of *p* = 0.0001 in the above stepwise regression analysis. However, to avoid multiple comparisons given the number of cases is 6 × 3 × 40 × 23 = 16,560, we did not consider isolated time-frequency regions (blue regions in Figure 2D). Namely, time-frequency domains that provided significant models including the identical factor of interest in consecutive time and frequency regions (yellow regions in Figure 2D) were considered a domain of STFM. The significance of the time-frequency region taken in this way was 0.0001 × (1 – 0.9999^8^) × 16,560 = 0.0013, indicating that a predictor variable that is significant in the time-frequency domain is sufficiently reliable.

## Results

The two monkeys exhibited high performance in the shape manipulation task (Sakamoto et al., 2012). We analyzed their behavioral performance during the shape manipulation task with minimal steps. In particular, we examined the reaction times (RTs) during 5 days of the training session. On average, Monkeys 1 and 2 performed 2,173 and 1,756 trials per day, respectively. Their success rates were 90% and 99%, and their minimum-step (two-step) success rates were 89% and 89%, respectively. The mean RTs for the first and second steps of the successful minimum-step trials were as follows: Monkey 1: 459 ± 257 ms (1st step), 510 ± 268 ms (2nd step); Monkey 2: 776 ± 264 ms (1st step), 684 ± 213 ms (2nd step).

Local field potentials were recorded from the dorsal and ventral sides of the principal sulcus of the left hemisphere of the LFPC during a shape manipulation task (Monkey 1, 32 locations; Monkey 2, 10 locations; Figure 2A), and their time-frequency modulations were analyzed by stepwise regression analysis. Although all task factors were used in the analysis as predictor variables, we focused on the two categories of factors related to presented shape and behavioral rules. In addition, because in a previous study (Sakamoto et al., 2020a) task-phase-dependent modulations of the theta and lower gamma components were well distinguished between the dorsal and ventral sides, we mainly discuss these frequency components below.

Representative examples from the ventral LFPC of the two monkeys are shown in Figure 3. In these examples, the predictor variable relevant to action planning, Transformed Shape in Sample (i.e., whether the sample shape is large or small in the sample period) appears to be reflected in the theta modulation during the delay period. Figures 3A,B show the differential time-frequency spectra between the cases in which the sample shape was large and small. From these, a large difference in low-frequency components during the delay period are observed in common. To confirm this, the raw LFP waveforms (Supplementary Figure 1, top) were filtered in the frequency range of interest to obtain their upper envelopes (Supplementary Figure 1, middle and bottom). In the averaged upper envelopes of these examples, persistent significant differences were observed in the theta range during the delay period when the sample shape was large (*p* = 1.3 × 10^–7^ at the largest difference, *t*-test: Figure 3C, bottom; *p* = 0.0020 at the largest difference, *t*-test: Figure 3D, bottom). Similar increases during the delay period were found for the delta range as well, but were not significantly greater in these cases (*p* = 0.048 at the largest difference, *t*-test: Supplementary Figure 2C, bottom; *p* = 0.026 at the largest difference, *t*-test: Supplementary Figure 2D, bottom). Large and clear differences in theta waves during the delay period shown above were not recognized in cases of rule-related predictor variables (Assignment, Figures 3G,H, bottom; Visual or Memory, Figures 3I,J, bottom) or the other shape-related variable, Shape Specific Attribute, which was not relevant to action planning (Figures 3E,F, bottom). In terms of the delta range, consistent modulations between the two examples were not seen, although some predictor variables exhibited significant modulations (Supplementary Figures 2E–J, bottom). This observation implies that theta and delta waves do not always show consistent modulations. Similarly, these two examples did not have common predictive variables causing many significant modulations in the lower and higher gamma ranges, although the numbers slightly differ depending on the variables (Figures 3C–J top, Supplementary Figures 2C–J top).

Typical examples of the dorsal side provide a different impression than the ones above. Differences in higher frequency ranges over the entire preparatory period in the differential spectra for Assignment, the rule the monkeys have to keep in mind during a certain trial block (Figures 4A,B), were observed. As in Figure 3, to observe this impression in detail, we obtained averaged upper envelopes, and examined the modulations in each predictor variable and frequency range of interest. As can be seen in Figures 4G,H (top), the lower gamma range exhibited frequent significant differences due to the predictor variable Assignment. In contrast to the ventral side, we did not recognize striking features common to the two examples in the gamma ranges (Figures 4C–J and Supplementary Figures 3C–J, top) for other predictor variables including another rule-related variable, Visual or Memory, which seems less burdensome to memorize than Assignment. In the low frequency ranges, the delta range showed slight modulation related to the task event, but not specific to any particular predictor variable. For the theta range, it did not show non-specific modulation in the initial hold and delay periods as seen in the ventral side (Figures 4C–J, bottom), except for significant differences that appeared intermittently in the right column examples only (Figures 4D,F,J, bottom). The examples shown in Figures 3, 4 suggest that task-dependent modulations of LFPs can distinguish the ventral side from the dorsal side. Task-relevant shape information, especially Transformed Shape in Sample Scale, was reflected in LFPs in the theta and lower frequency bands during the delay period on the ventral side while Assignment, the rule to be kept in mind, influenced the overall increase in gamma waves on the dorsal side.

Among the shape- and rule-related predictor variables, Transformed Shape in Sample Scale variable had a prominent number of domains exhibiting STFM, followed by Assignment (Supplementary Figure 4A). Each of the other shape- and rule related predictor variables, such as Transformed Shape in Test, which had not been expected to cause LFP modulations during the preparatory period, and Shape-Specific Attribute Round or Sharp, which is not relevant to task execution, had a small number of STFM domains and no characteristic frequency distribution (Supplementary Figures 4C,D). Therefore, the following discussion will focus primarily on Transformed Shape in Sample and Assignment.

**FIGURE 5:**
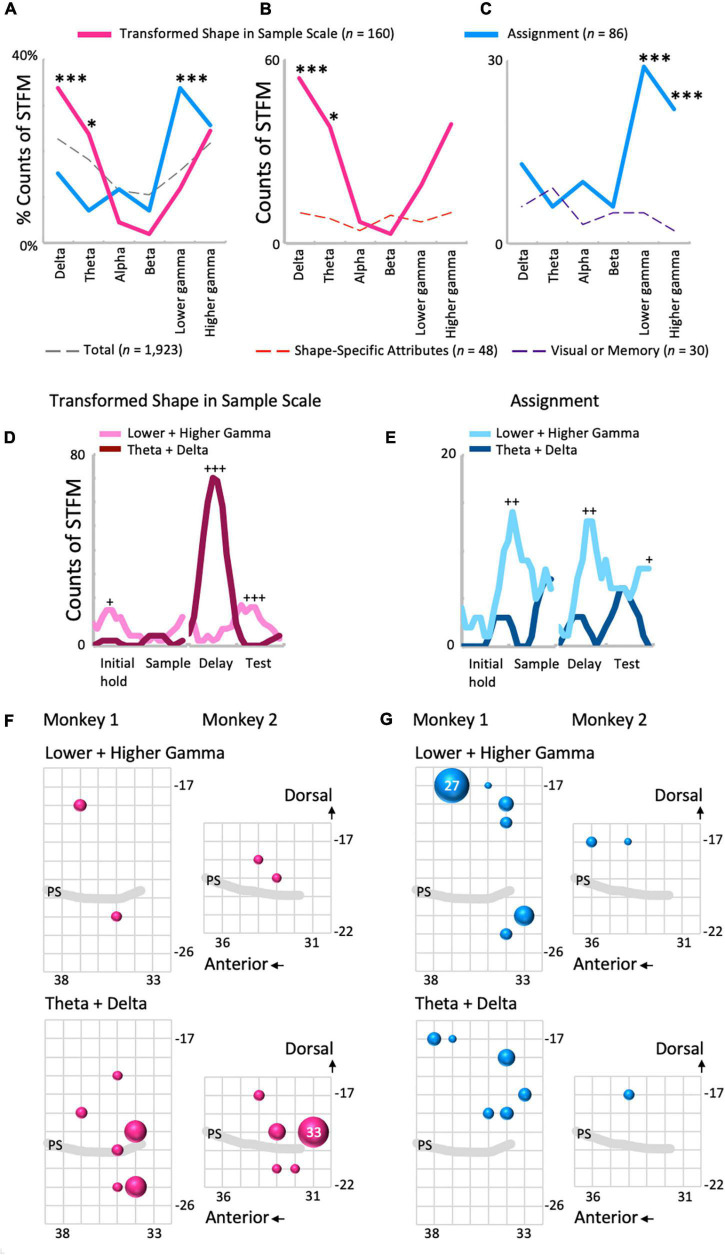
Comparisons between the influence of shape- and rule-related predictor variables on LFP modulations. **(A)** The frequency distributions of the proportions of STFMs for Transformed Shape in Sample Scale **(B)**, Assignment, and all variables (*n* = 23). **(B,C)** The frequency distributions of the count of STFMs for Transformed Shape in Sample Scale **(B)** and Assignment **(C)** compared to each control. *p < 0.05, ^***^p < 0.001. **(D,E)** Time-developments of the counts of STFMs for the lower gamma and theta ranges. Transformed Shape in Sample Scale **(D)** and Assignment **(E)**. +*p* < 0.005, ++*p* < 0.001, +++*p* < 0.0001. **(F)** Spatial distributions of the STFMs of gamma (top) and theta-delta (bottom) ranges for Transformed Shape in Sample Scale during the delay period. The largest circle represents *n* = 33. **(G)** Same plots for Assignment. Counts are the summation of the entire preparatory period. The largest circle represents *n* = 27.

Local field potentials modulation by Transformed Shape in Sample was common in the lower frequency range (Figures 5A,B and Supplementary Figure 4B), while STFM by Assignment was seen mainly in higher frequencies (Figures 5A,C). The percentages of the counts of the STFM domains exhibiting along the frequency range are shown in Figure 5A. In the percentage distribution of STFM domains for all predictor variables (*n* = 1,923; Figure 5A, dashed gray line), the proportions in the low-frequency (theta and delta) and high-frequency (higher and lower gamma) regions were high, and those in the mid-frequency (beta and alpha) region were low. The Transformed Shape in Sample: Scale trend was significantly emphasized in the low-frequency region compared to the above-mentioned total distribution (theta: *p* = 0.043; delta: *p* = 0.00086, binomial test: Figure 5A, red line). By contrast, for Assignment, the distribution in the high-frequency region, particularly in the lower gamma region, was more pronounced (*p* = 3.1 × 10^–5^, binomial test: Figure 5A, blue line). The frequency distribution of the counts of STFM by Transformed Shape in Sample Scale was higher in the low frequency region than expected from the percentage distribution of Shape-Specific Attributes (theta: *p* = 0.013; delta: *p* = 0.00010, binomial test: Figure 5B). On the other hand, the counts of STFM by Transformed Shape in Sample Scale were prominent only in the delta range (Supplementary Figure 4B). By contrast, the counts of STFM for Assignment were higher in the high frequency range compared to the expected values from Visual or Memory (higher gamma: *p* = 3.4 × 10^–8^; lower gamma: *p* = 8.9 × 10^–5^, binomial test: Figure 5C).

The time distribution of the counts of STFM, obtained using a sliding time-window of 500 ms to sum, also contrasted between these two predictor variables (Figures 5D,E and Supplementary Figure 5). As for Transformed Shape in Sample Scale, gamma modulations counts exceeded that of theta-delta modulations in the initial hold (*n* = 15, *p* = 0.0012, binomial test) and test periods (*n* = 16, *p* = 1.5 × 10^–5^, binomial test), but the theta-delta modulations increased compared with the gamma modulations in the delay period (*n* = 70, *p* = 5.6 × 10^–19^, binomial test: Figure 5D). The excess of gamma modulations in the initial hold and test periods appear to be primarily due to the high gamma range (Supplementary Figure 5A), while both the theta and delta range exhibited a consistent increase in STFM counts in the delay period (Supplementary Figure 5B). On the other hand, the gamma modulations by Assignment were maximal in the early sample presentation period compared with the theta-delta range (*n* = 14, *p* = 0.00049, binomial test), but had other significant peaks in the delay (*n* = 13, *p* = 0.00092, binomial test) and test periods (*n* = 8, *p* = 0.0039, binomial test), and even started to increase from the late initial hold period (Figure 5E). This overall increase in STFM counts in the gamma range during the preparatory period cannot be clearly attributed to either higher or lower gamma (Supplementary Figure 5C). For the lower frequency range, the STFM counts of delta, but not theta, increased during the test period, although not significantly (Supplementary Figure 5D).

Spatial distributions of the STFM counts were also distinguishable between these two predictors. Transformed Shape in Sample Scale had many STFM counts in the theta-delta range on the ventral side of the recorded area (Figure 5F) while for Assignment, the STFM counts in the dominant gamma and theta-delta ranges were biased to the dorsal side (Figure 5G). The spatial distributions shown in Figures 5F,G were tested using the Wilcoxon rank sum test. As shown in Table 2, the theta-delta distributions of Transformed Shape in Sample Scale are significantly biased to the ventral side compared to both the theta-delta and gamma distributions of Assignment. The gamma and theta-delta distributions of Transformed Shape in Sample Scale were also distinguished along the anterior-posterior axis. These spatial biases were observed consistently in both monkeys. In the theta-delta map of Transformed Shape in Sample Scale, STFM counts are seen just above the principal sulcus. However, as long as they are not far from the principal sulcus, they are considered to have been obtained from the ventral LFPC. The LFP data analyzed in this study were obtained from the bottom-most contact (ch.15) of a U-probe. When the U-probe penetrated the convex or the cortex almost perpendicularly to its surface, the time-frequency spectra of LFPs was similar across all contacts (Supplementary Figure 6). By contrast, when it was inserted into a site dorsal to the principal sulcus, the spectra changed largely along the U-probe shank (Supplementary Figure 7). Taking into consideration that the principal sulcus runs in the medial direction (Paxinos et al., 1999; Sakamoto et al., 2015), the LFPs obtained from the lower contacts were expected to be located in the ventral bank of the principal sulcus. This dorsal-ventral distinction was not prominent in other predictive variables in this study (Supplementary Figure 8). For Transformed Shape in Sample Rotation, a significant number of STFM can be seen in the ventral side. However, some STFM counts were also seen in the dorsal side (Supplementary Figure 8A). The variable Shape-Specific Attribute Isosceles or Right Angle provided scattered distributions in both sides of the principal sulcus (Supplementary Figure 8B). Distribution of the rule-related variable Visual or Memory appeared biased to the dorsal side, although this unclear when compared to Assignment.

**TABLE 2:** Wilcoxon rank-sum test between the STFM distributions shown in Figures 5F,G.

Medial vs. Lateral (Monkey1)					
		**Transformed shape in sample scale**		**Assignment**	
		**Lower + higher gamma**	**Theta + delta**	**Lower + higher gamma**	**Theta + delta**
Transformed shape in sample-scale	Lower + higher gamma	*p* =	0.065	0.08	0.39
	Theta + delta			0.00000032	0.00000015
Assignment	Lower + higher gamma				0.16
	Theta + delta				

**Medial vs. Lateral (Monkey2)**					
		**Transformed shape in sample scale**		**Assignment**	
		**Lower + higher gamma**	**Theta + delta**	**Lower + higher gamma**	**Theta + delta**

Transformed shape in sample scale	Lower + higher gamma	*p* =	0.0412	0.0571	0.13
	Theta + delta			0.00032	0.0025
Assignment	Lower + higher gamma				1.0
	Theta + delta				

**Anterior vs. Posterior (Monkey1)**					
		**Transformed shape in sample scale**		**Assignment**	
		**Lower + higher gamma**	**Theta + delta**	**Lower + higher gamma**	**Theta + delta**

Transformed shape in sample scale	Lower + higher gamma	*p* =	0.0021	0.42	0.08
	Theta + delta			0.040	0.85
Assignment	Lower + higher gamma				0.35
	Theta + delta				

**Anterior vs. Posterior (Monkey2)**					
		**Transformed shape in Sample Scale**		**Assignment**	
		**Lower + higher gamma**	**Theta + delta**	**Lower + higher gamma**	**Theta + delta**

Transformed shape in Sample Scale	Lower + higher gamma	*p* =	0.0046	0.17	0.80
	Theta + delta			0.0015	0.013
Assignment	Lower + higher gamma				0.6

## Discussion

We recorded LFPs from the monkey LFPC during a shape manipulation task and analyzed its task-dependent modulations through wavelet analysis and stepwise regression analysis. Here, we mainly focused on shape- and rule-related task variables (Sakamoto et al., 2020a), and found that the transformed shape in the sample period strongly affected the theta and delta waves in the delay period on the ventral side, and the arm-manipulation assignment influenced gamma components on the dorsal side.

Our results are sufficiently reliable for the following reasons. First, we analyzed LFPs recorded from the bottommost contacts of multi-contact electrodes, and the characteristic task-related LFP modulations at each recording site were consistent, at least at adjacent sites (Supplementary Figures 6, 7). Second, using stepwise regression analysis to generate the best explanatory model from all predictor variables, we were able to avoid spurious models; the difference wavelet spectrum for the Assignment variable shown in Figure 4A (left column) shows that differences in the gamma and beta ranges are emphasized. If we had performed a simple regression analysis with each single predictor variable for such data, it would have indicated Assignment as a significant predictor variable for beta waves as well. However, because of the stepwise regression analysis, it was clear that this beta component reflects a trial that repeats the same conditions as the previous trial.

The preponderance of LFP modulations by shape-related information on the ventral side in our results was consistent with previous findings. With respect to visual shape-related processing, the ventral LFPC is anatomically characterized by reciprocal connections with the inferior temporal (IT) cortex (Ungerleider et al., 1989; Webster et al., 1994). Reflecting this, neuronal activities related to visual shape have been reported (Ninokura et al., 2004), including activities in delay period (Wilson et al., 1993; Freedman et al., 2002, 2003). In the delayed matching-to-category task of Freedman et al. (2002, 2003), monkeys were required to forcibly categorize an intermediate sample shape obtained by morphing to one of two test shapes presented after delay. During this task, unit activities influenced by the continuous changes of shape-specific attributes were observed in the IT cortex, while activities reflecting the task requirement, i.e., two-choice categorization during the delay period were seen in the ventral LFPC. These delayed activities may be involved not only in retention of sample information, but also in anticipation or recall of upcoming test stimuli. This aspect of anticipation or recall is also common to the pair-association memory task that has long been used to study the IT cortex (Sakai and Miyashita, 1991). Recently, it has been reported that, during this task, LFP theta waves are generated in the temporal lobe (Koyano et al., 2016; Nakahara et al., 2016). Especially, Koyano et al. (2016) showed that through the processing triggered by a sample stimulus within the cortical six-layer neural circuit, spiking activities reflecting the association of the upcoming test stimulus are generated in the output layer or layer 6, and that these cellular activities are synchronized to the LFP theta rhythm. Considering these findings, the theta waves observed in the ventral LFPC in this study are likely involved in anticipating the upcoming test stimulus as well as retaining the task-relevant shape information of the sample stimulus although interactions with the IT cortex.

As discussed above, predictor variables more relevant to planning and executing the shape manipulation task appear to cause more LFP modulations. In the present study, the shape transformation information during the sample period, which is directly related to behavioral planning, resulted in more STFM domains than the variables of shape-specific attributes. Moreover, among shape-specific attributes, the macroscopic feature isosceles or right triangles (Figure 2B) had more STFM domains than the microscopic feature round or sharp corners (Figure 2C), which presumably reflects the importance of recognizing macroscopic features as a basis for understanding the transformation between sample and test shapes. These observations suggest that LFPs play an important role in adaptive coding in the prefrontal cortex (Duncan, 2001). Furthermore, the fact that the task-relevant information needed to be retained in working memory in our task and the theta modulation during the delay period was correlated with this behavioral demand is consistent with previous studies suggesting that task-relevant information in the prefrontal short-term memory is stored through theta-range synchronization with other cortical areas (Liebe et al., 2012).

It remains unclear why, among the variables for shape transformation information during the sample period, rotation exhibited fewer STFM domains than scale (Supplementary Figure 4A). The scattered spatial distribution of STFM domains for rotation during the sample period (Supplementary Figure 8A) may indicate that the working memory for rotation could be stored in a distributed manner, i.e., in both the ventral and dorsal sides. Given the involvement of the dorsal side in spatial working memory, the above observation may imply that the monkeys memorize the rotation information not only as shape transformation but also as the spatial location of particular features of the shape.

The theta-range properties described above were similarly observed in delta waves. While delta waves are widespread in the brain during slow-wave sleep, delta-range synchronization between cortical areas associated with decision making has been reported in awake monkeys (Nácher et al., 2013). Given that information held in working memory is used to make behavioral decisions in our task, the enhanced delta waves seen in the present study may also be involved in decision-making and its associated communication with other brain areas.

More LFPs were modulated by assignment in the dorsal side, which is consistent with the neuronal activity reflecting retrieval of the task-related information in this side (Hoshi and Tanji, 2004). However, in the LFPC during the path-planning task we used in previous studies, which also required switching between motor and operation assignments, the changes in neuronal firing in response to the assignments were less pronounced (Saito et al., 2005). We have not yet analyzed the properties of neuronal firings in this shape manipulation task, but it is possible that the motor-operation assignment affects LFPs and spiking activities differently. The LFP modulation was largest at the beginning of the sample period, but was also large from the beginning of the trial, likely because the task is performed in blocks and it is necessary to keeping the current assignment in mind during the same block. On the other hand, there were few LFP modulations corresponding to the other rule variable, visual or memory. In the visually guided conditions, the contour of the sample period shape was superimposed on the test shape, and thus it was not necessary to remember the transformed shape presented in the sample period and was expected that there would be significant LFP modulation reflecting this difference in task requirements. However, this was not the case. The observed smaller difference between visual and memory conditions may be because it is advantageous to understand the shape presented in the sample period from the beginning of the trial to perform the task robustly even in the visually guided trials. Alternatively, considering the distinct contributions of the premotor cortex to the generation of visually guided sequential behavior and the supplementary motor cortex to the generation of memory-guided sequential behavior (Mushiake et al., 1991), there may be other areas responsible for the changes in activity associated with the difference in visually and memory-guided trials.

The distinction between ventral and dorsal sides by LFPs shown in this study is consistent with previous studies on neuronal firings, as described above. However, depending on the experimental setup and task factors, it is possible that neither cell firings nor LFP modulations distinguish between ventral and dorsal sides, because dorsal and ventral cells share common inputs (see Tanji and Hoshi, 2008). If the task contains only factors associated with the common inputs, it is natural that the two are not distinguished. As shown in Supplementary Figure 8, the LFP modulations by other predictive variables analyzed in this study did not distinguish between ventral and dorsal sides well. It is about the“sharpness” of the task; i.e., the fact that neural activity during a task does not distinguish between two areas does not mean that there are no functional differences between the two areas. It should be recognized that the area called Broadman’s 6 was not distinguished until the neural activities during the task requiring visually induced or memory induced generation of sequential actions distinguished premotor and supplementary motor areas within this area (Mushiake et al., 1991). Our results indicate that the gamma and theta waves reflected the information of assignment and transformed shape in Sample, respectively; this is consistent with the view of Roux and Uhlsaas (2014), who proposed that gamma-band waves are generally involved in the maintenance of working memory information, whereas theta-band waves underlie the organization of sequentially ordered working memory items. In our case, the assignment information needed to be firmly maintained during the corresponding block of trials. By contrast, the information of the transformed shape in Sample was temporally stored and used to plan sequential actions.

Wutz et al. (2018) analyzed LFPs in the LFPC during the execution of a dot-pattern categorization task and observed a gamma wave modulation corresponding to less abstract categories in the ventral side and beta wave modulation corresponding to more abstract categories in the dorsal side. The involvement of gamma waves in retaining the dot pattern in working memory for discrimination after a delay period is consistent with the ideas regarding gamma waves discussed above. On the other hand, the interpretation of beta wave modulation as corresponding to complex categories needs to be revisited. Beta waves are often found in cortical motor-related areas (Murthy and Fetz, 1992; Hosaka et al., 2016), and these observations evoke the idea that beta waves are related to the readiness of behavior. In Wutz et al. (2018), animals were to choose between two stimuli that belonged to the same category as the sample stimulus, with free viewing during the choice period. In this task structure, after presentation of the sample, the animals prepare a series of visual search eye-movements to identify the stimulus that belongs to the same category as the sample. The LFPC includes neural activities corresponding to cognitive (Cromer et al., 2010) and behavioral (Shima et al., 2007; Sakamoto et al., 2020b) categories, which are sometimes indistinguishable. Noton and Stark (1971) demonstrated that visual concepts exist in the brain as stylized relationships between features through an experiment in which subjects were shown a large visual image moved their gaze around the features to identify the image. The beta waves reported by Wutz et al. (2018) may reflect such cognitive and behavioral readiness.

Why does functional differentiation exist in the LFPC? Functional differentiation is found in many aspects of the nervous system. For example, organs specialized to detect light provide information essential for survival. However, is it also necessary to have something not specialized in a particular function to cope with various situations? For example, computers can be used for a variety of purposes because they are not specialized for any particular use. Is it important for the LFPC as a center of executive functions to specialize in a particular function? The results of this study refute this notion. Rather, they imply that functionally different parts can work together to create novel ideas when faced with unknown situations; in our society, innovations happen when individuals with different personalities work cooperatively. LFPs and their synchronization may also play an important role in this“cooperation.”

## Data Availability Statement

The raw data supporting the conclusions of this article will be made available by the authors, without undue reservation.

## Ethics Statement

The animal study was reviewed and approved by the Animal Care and Use Committee of Tohoku University.

## Author Contributions

KS and HM designed the research. KS and NK performed the research. KS analyzed the data, wrote the first draft of the manuscript, edited and wrote the manuscript. All authors contributed to the article and approved the submitted version.

## Conflict of Interest

The authors declare that the research was conducted in the absence of any commercial or financial relationships that could be construed as a potential conflict of interest.

## Publisher’s Note

All claims expressed in this article are solely those of the authors and do not necessarily represent those of their affiliated organizations, or those of the publisher, the editors and the reviewers. Any product that may be evaluated in this article, or claim that may be made by its manufacturer, is not guaranteed or endorsed by the publisher.

## Supplementary Material

Supplementary Figure 1 A depiction of how upper envelops in Figures 3, 4 were obtained. Typical LFP waveforms obtained when a “large” (orange) or “small” (blue) sample shape was presented. Top: raw waveforms. Middle: lower-gamma waves (30–60 Hz) and their upper envelopes. Bottom: theta waves (3–7 Hz) and their upper envelopes.

Supplementary Figure 2 Waveforms in the delta and high gamma range obtained from the same recording site as in Figure 3. **(A–J)** Each panel is identical to the corresponding panel in Figure 3.

Supplementary Figure 3 Waveforms in the delta and high gamma range obtained from the same recording site as in Figure 4. **(A–J)** Each panel is identical to the corresponding panel in Figure 4.

Supplementary Figure 4 Distributions of STFM domains across the predictor variables analyzed in this study **(A)** and frequency ranges **(B–D)**. Relations to Figures 5B,C are also indicated.

Supplementary Figure 5 Comparisons between the influence of shape **(A,B)** and rule-related **(C,D)** factors on LFP modulations. Time-developments of the counts of STFMs is shown for the higher and lower gamma **(A,C)**, and theta and delta **(B,D)** ranges. **(A)** Formats are the same as in Figures 5D,E. A sliding window of 500 ms was used. +*p* < 0.005.

Supplementary Figure 6 The differential time-frequency spectra of the transformed shape in sample period (large – small) obtained from the channels along the multi-contact electrode inserted into a site in the convexity of the ventral LPFC of Monkey 1 (AP 34, DV –25). The spectra of ch.15, circled in yellow, are identical to those in Figure 3A and Supplementary Figure 2A.

Supplementary Figure 7 Spectra in the same format as Supplementary Figure 6 obtained from the electrode inserted into a site dorsal to the principal sulcus of Monkey 2 (AP 33, DV -19). Data from ch.15, circled in yellow, are included in the analysis. Note that although these spectra exhibited large changes compared to those in Supplementary Figure 6, the lower channels showed spectra similar to that of ch.15, suggesting that the data from the lower channels were obtained from the ventral LPFC.

Supplementary Figure 8 Spatial distributions of STFM domains for Transformed Shape in Sample Rotation **(A)**, Shape-Specific Attributes Isosceles or Right Rectangle **(B)**, and Visual or Memory **(C)**. The scale of the balls in **(A,B)** of shape-related variables is proportional to that in Figure 5F, and that in rule-related **(C)** is proportional to that in Figure 5G.
