# Supplementary figures and images for: Shape and Rule Information Is Reflected in Different Local Field Potential Frequencies and Different Areas of the Primate Lateral Prefrontal Cortex
Source: Front Behav Neurosci. 2022 May 13;16:750832. doi: 10.3389/fnbeh.2022.750832 (PMC9137426; doi:10.3389/fnbeh.2022.750832)

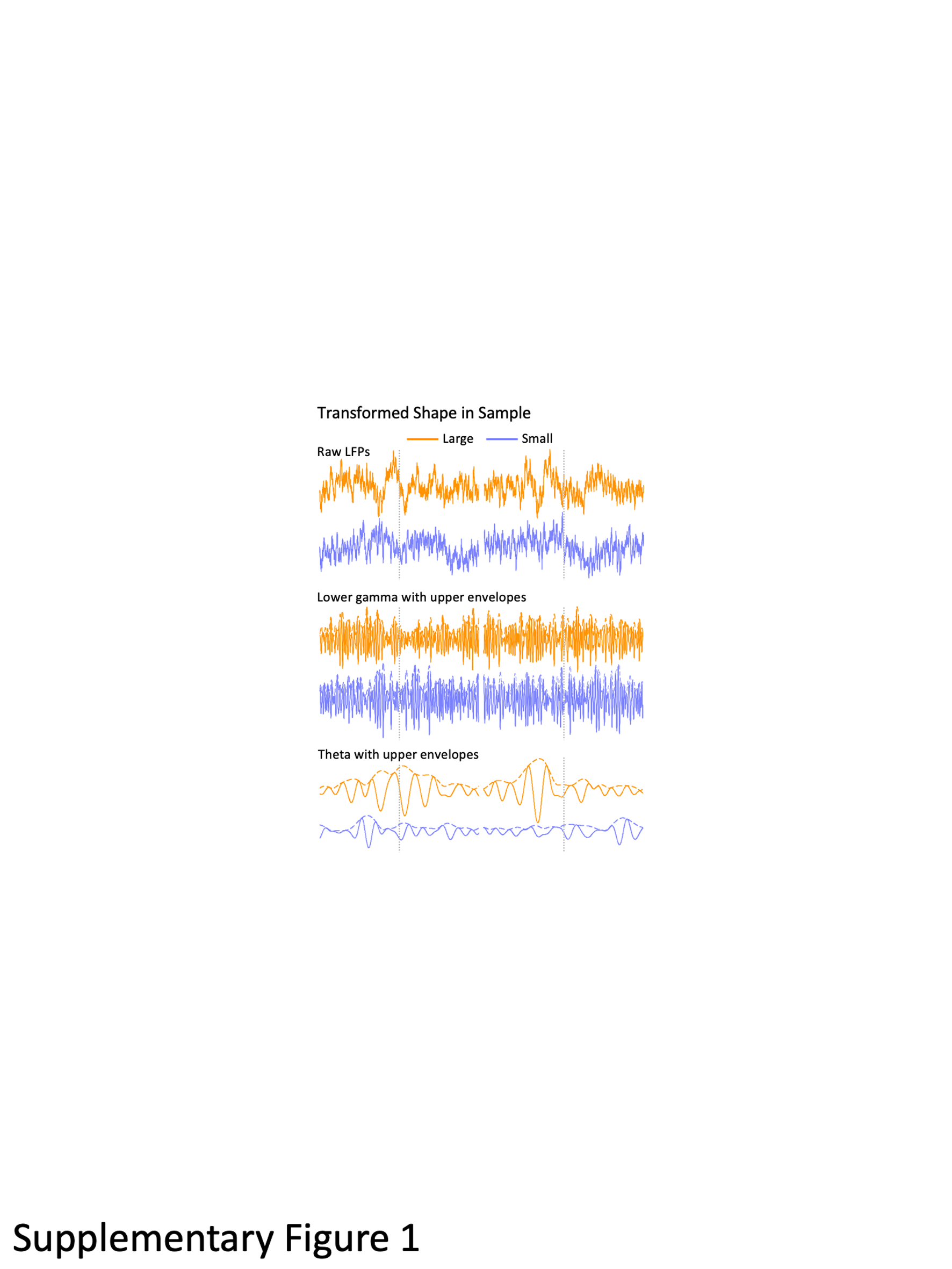

Supplement: Supplementary Figure 1 — A depiction of how upper envelops in Figures 3, 4 were obtained. Typical LFP waveforms obtained when a “large” (orange) or “small” (blue) sample shape was presented. Top: raw waveforms. Middle: lower-gamma waves (30–60 Hz) and their upper envelopes. Bottom: theta waves (3–7 Hz) and their upper envelopes. [file Image_1.TIFF]

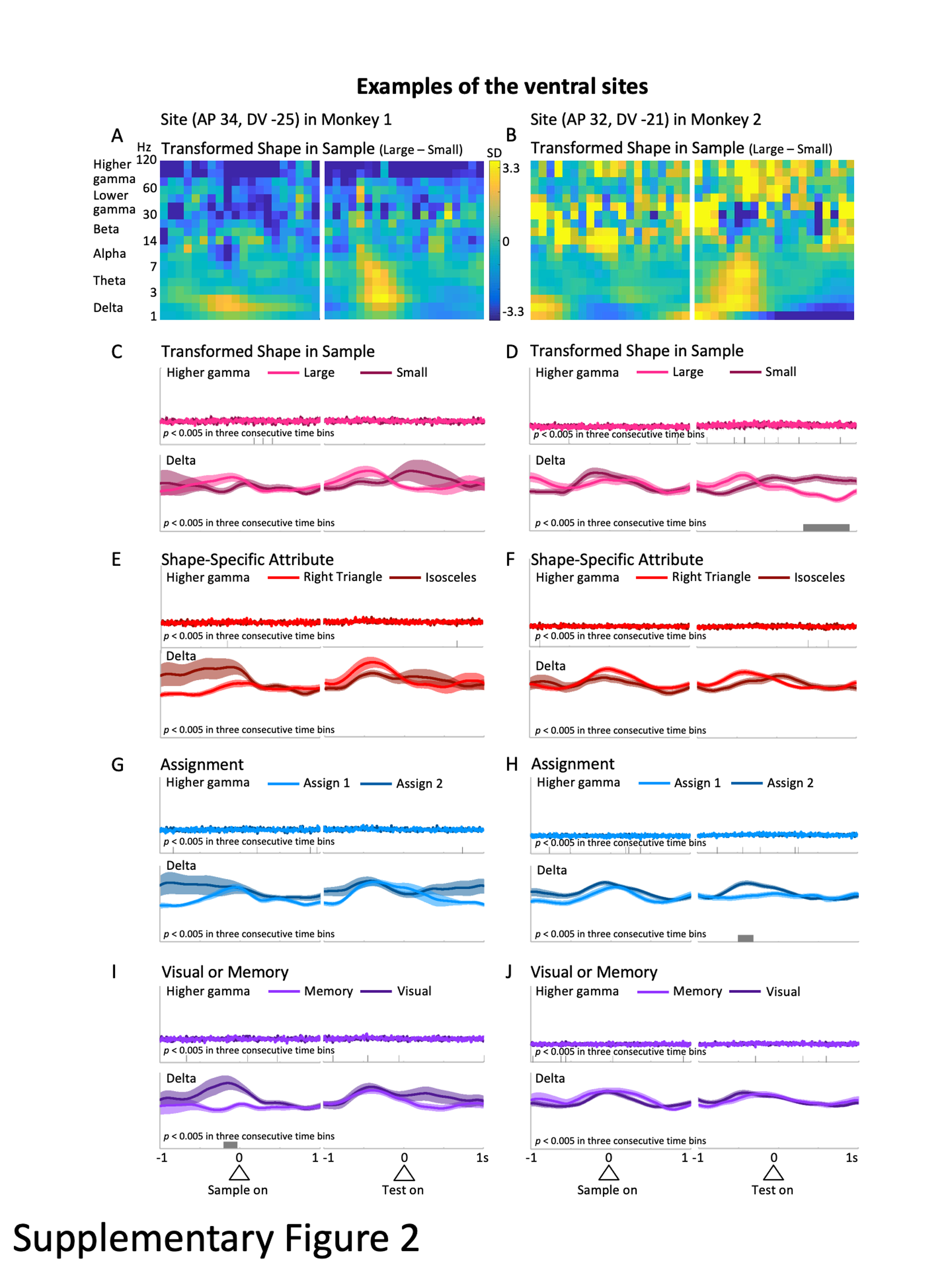

Supplement: Supplementary Figure 2 — Waveforms in the delta and high gamma range obtained from the same recording site as in Figure 3. (A–J) Each panel is identical to the corresponding panel in Figure 3. [file Image_2.TIFF]

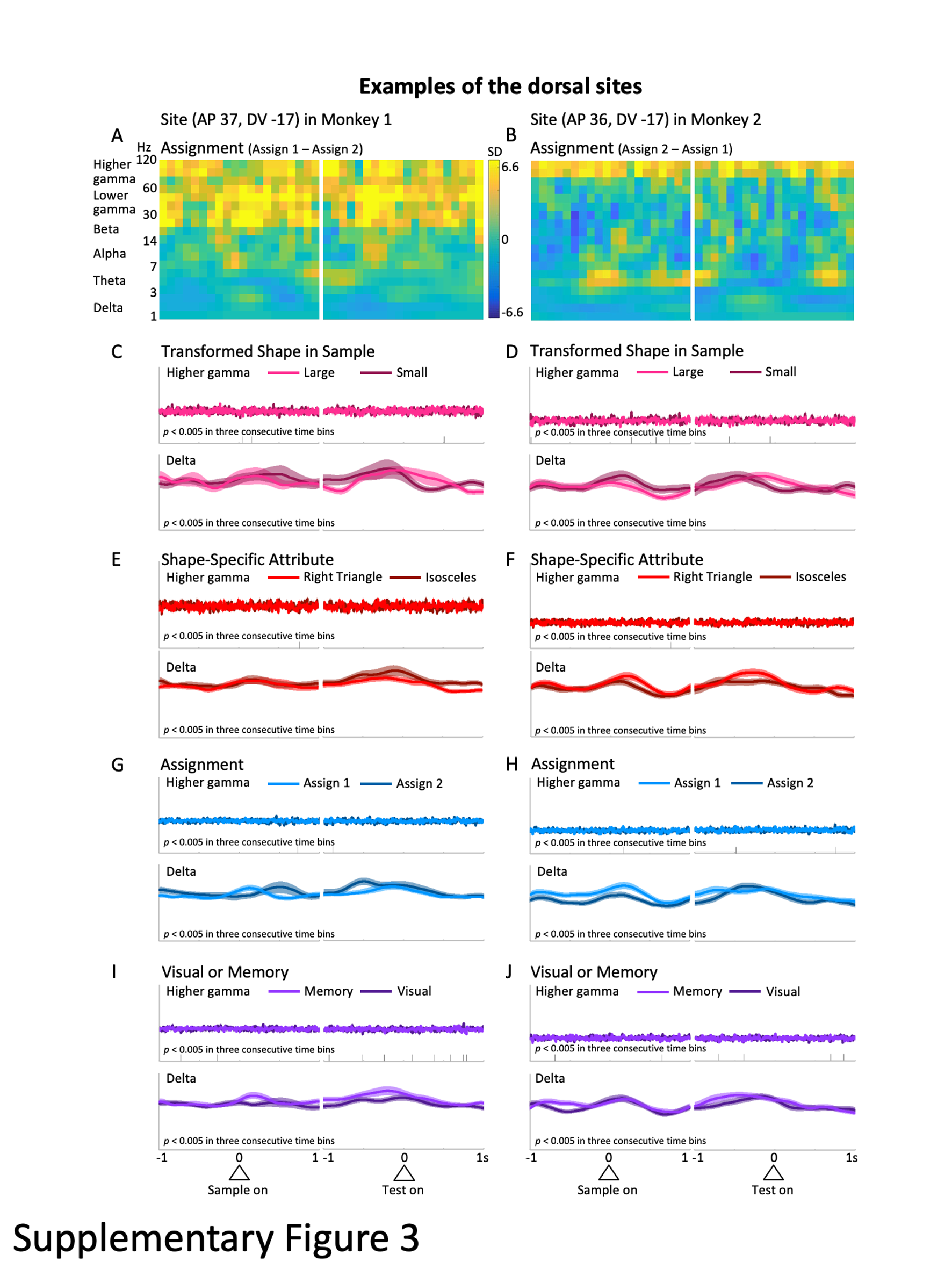

Supplement: Supplementary Figure 3 — Waveforms in the delta and high gamma range obtained from the same recording site as in Figure 4. (A–J) Each panel is identical to the corresponding panel in Figure 4. [file Image_3.TIFF]

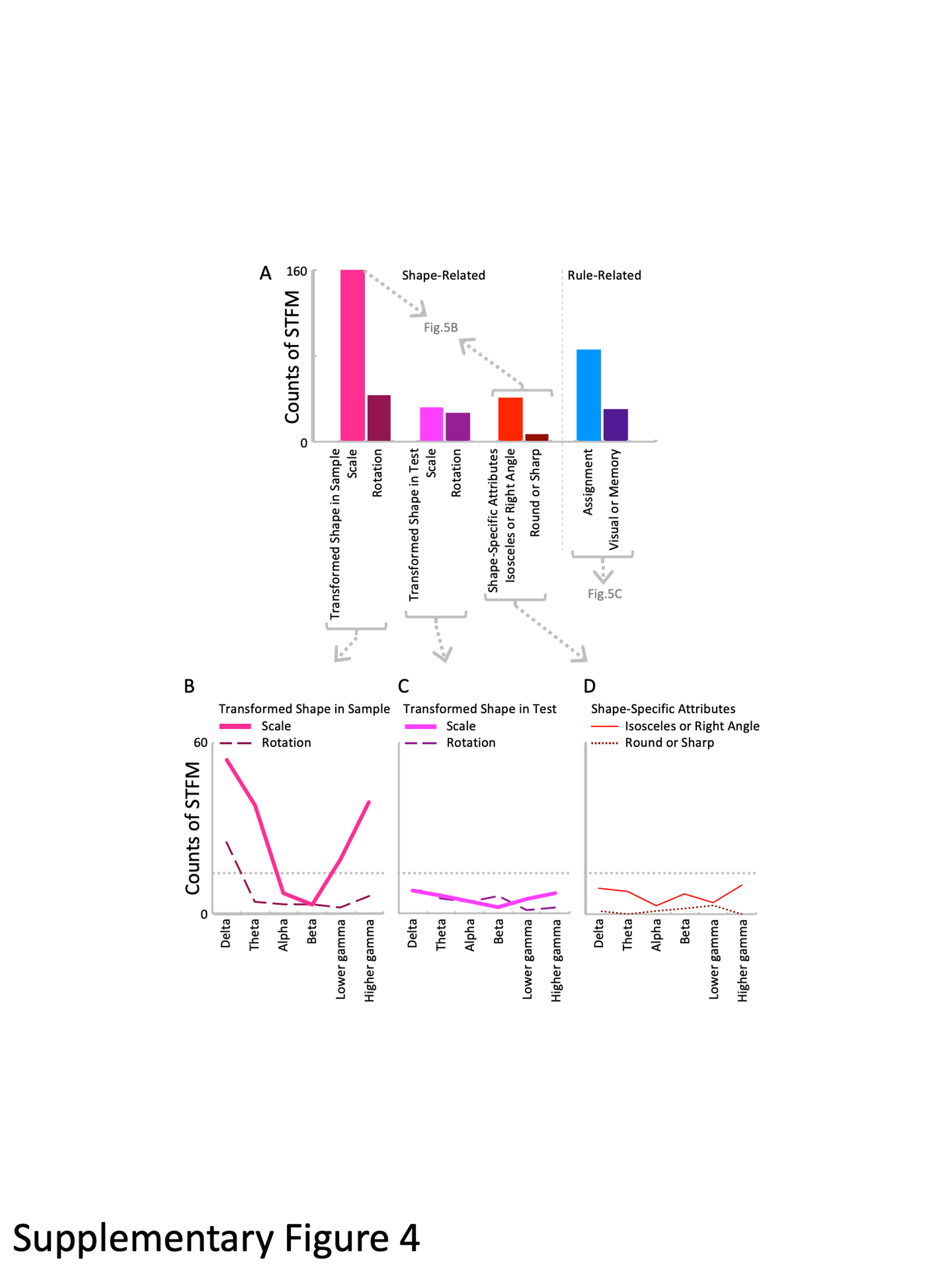

Supplement: Supplementary Figure 4 — Distributions of STFM domains across the predictor variables analyzed in this study (A) and frequency ranges (B–D). Relations to Figures 5B,C are also indicated. [file Image_4.TIFF]

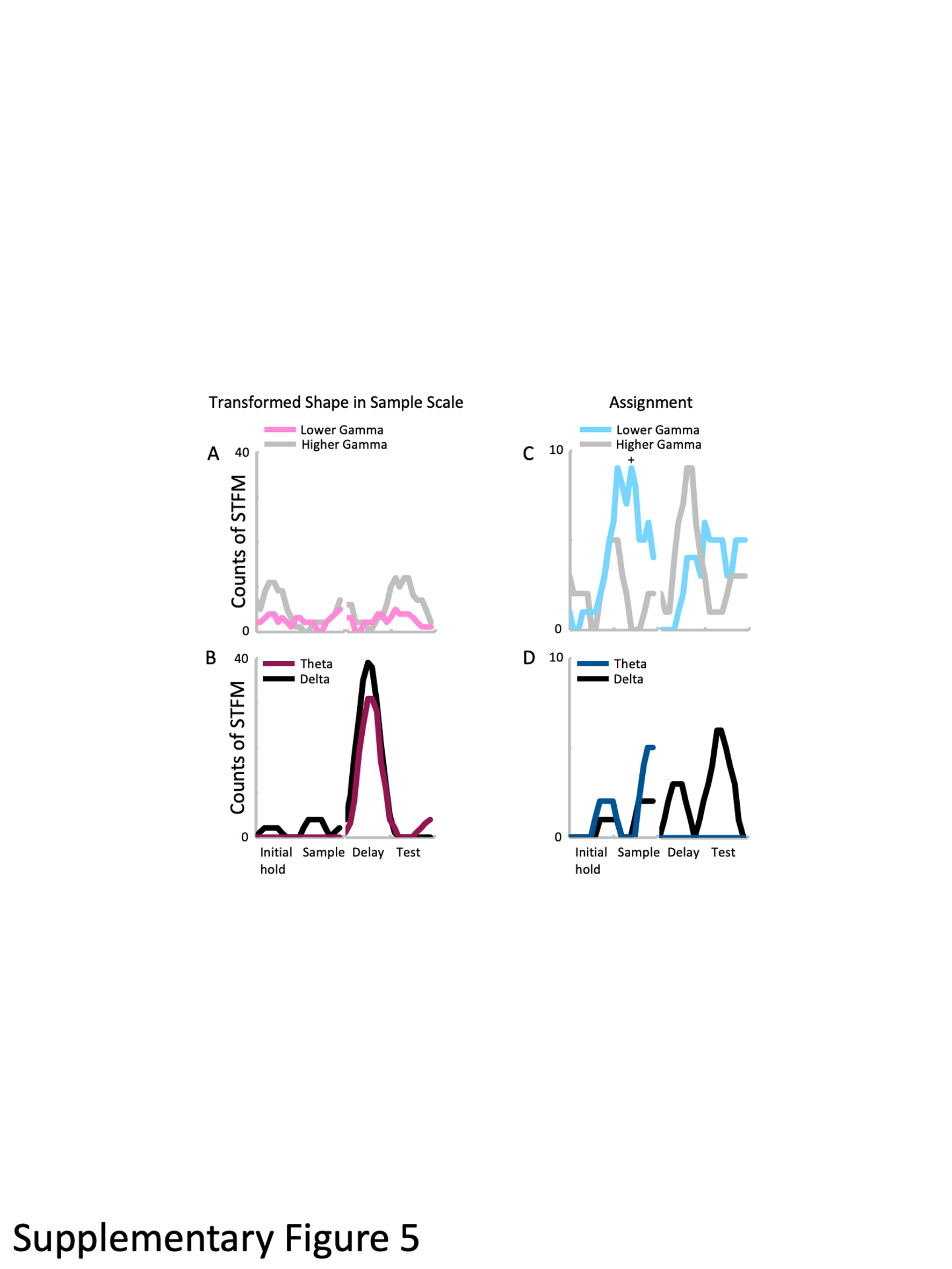

Supplement: Supplementary Figure 5 — Comparisons between the influence of shape (A,B) and rule-related (C,D) factors on LFP modulations. Time-developments of the counts of STFMs is shown for the higher and lower gamma (A,C), and theta and delta (B,D) ranges. (A) Formats are the same as in Figures 5D,E. A sliding window of 500 ms was used. +p < 0.005. [file Image_5.TIFF]

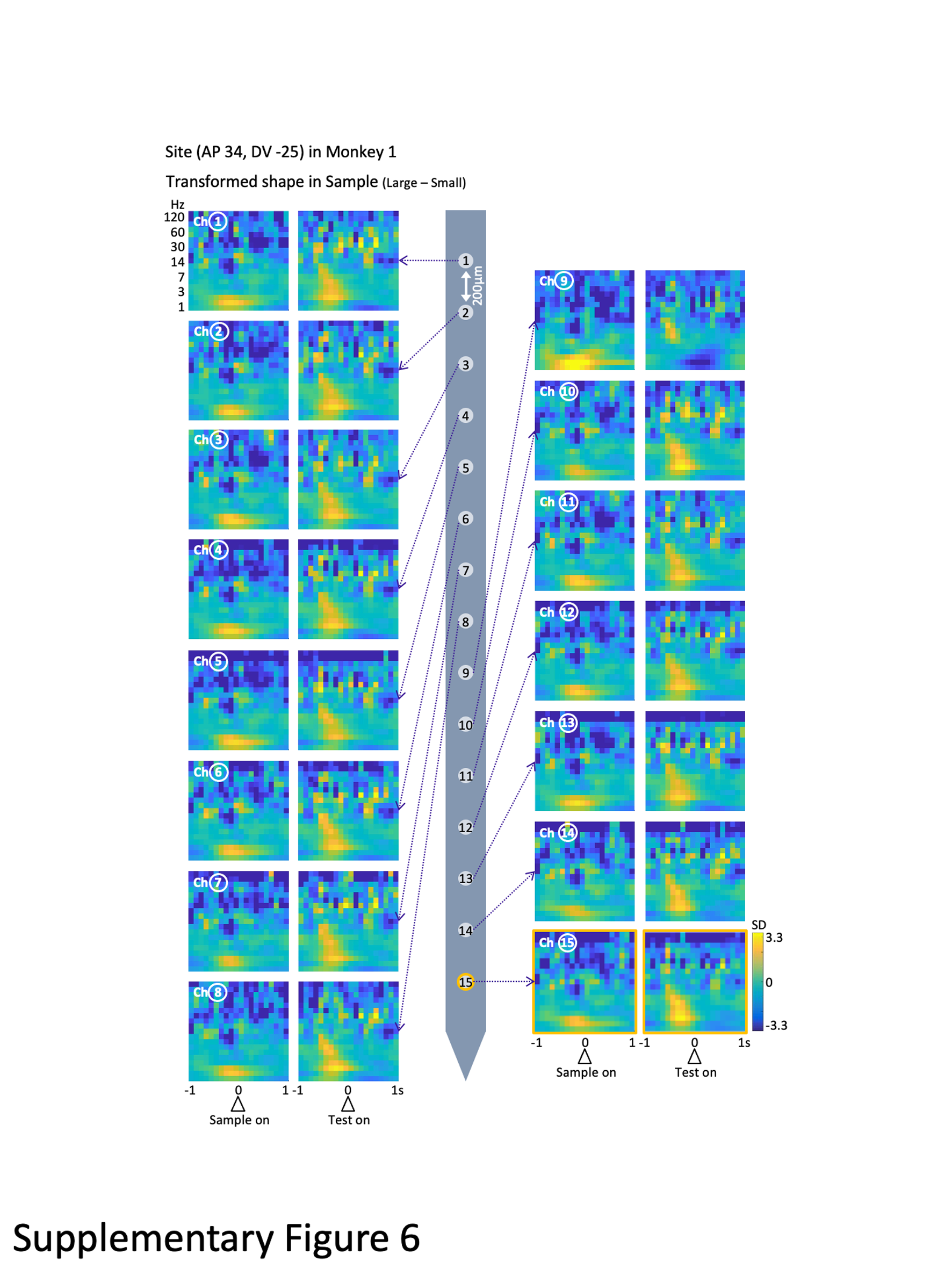

Supplement: Supplementary Figure 6 — The differential time-frequency spectra of the transformed shape in sample period (large – small) obtained from the channels along the multi-contact electrode inserted into a site in the convexity of the ventral LPFC of Monkey 1 (AP 34, DV –25). The spectra of ch.15, circled in yellow, are identical to those in Figure 3A and Supplementary Figure 2A. [file Image_6.TIFF]

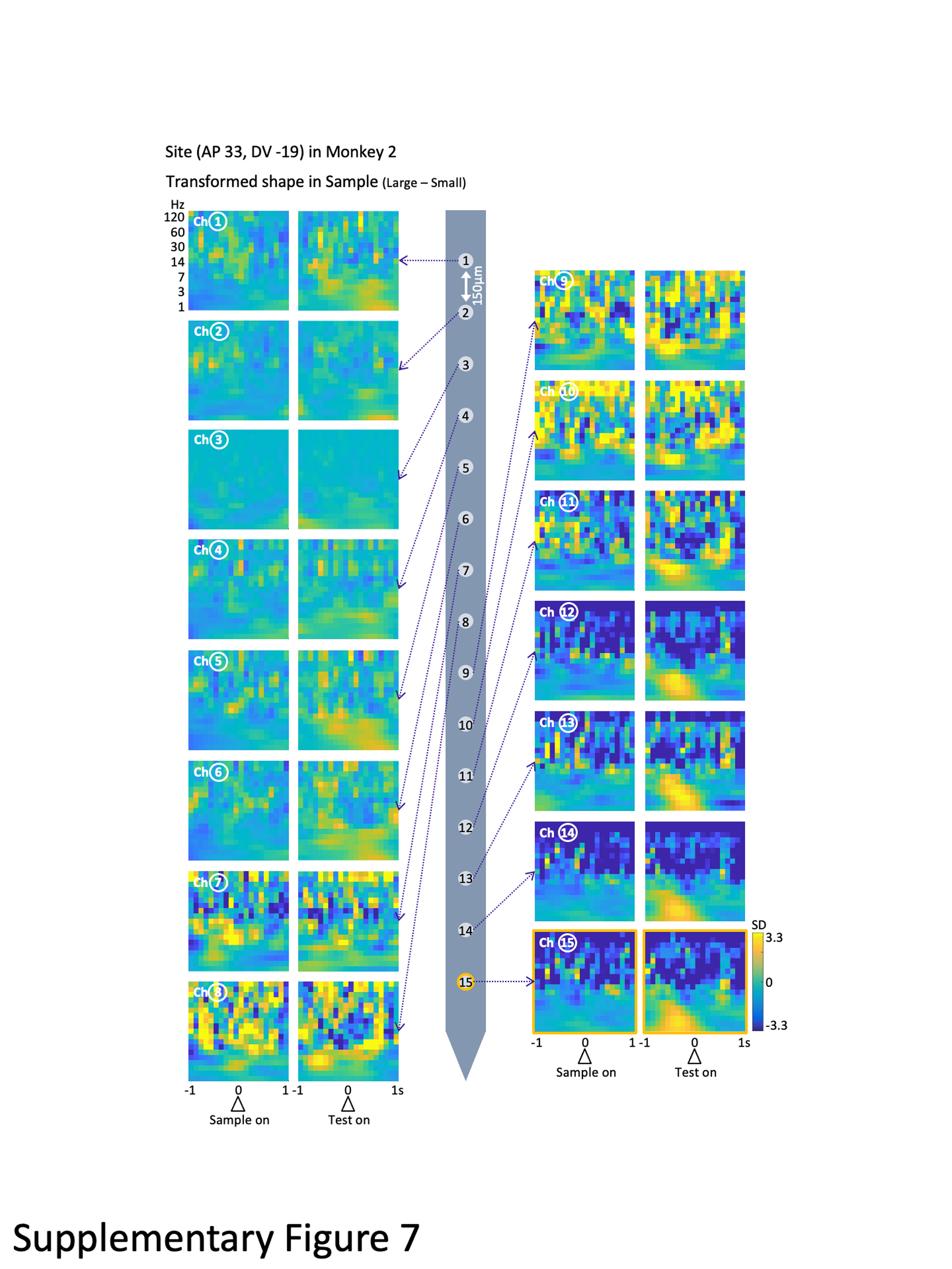

Supplement: Supplementary Figure 7 — Spectra in the same format as Supplementary Figure 6 obtained from the electrode inserted into a site dorsal to the principal sulcus of Monkey 2 (AP 33, DV -19). Data from ch.15, circled in yellow, are included in the analysis. Note that although these spectra exhibited large changes compared to those in Supplementary Figure 6, the lower channels showed spectra similar to that of ch.15, suggesting that the data from the lower channels were obtained from the ventral LPFC. [file Image_7.TIFF]

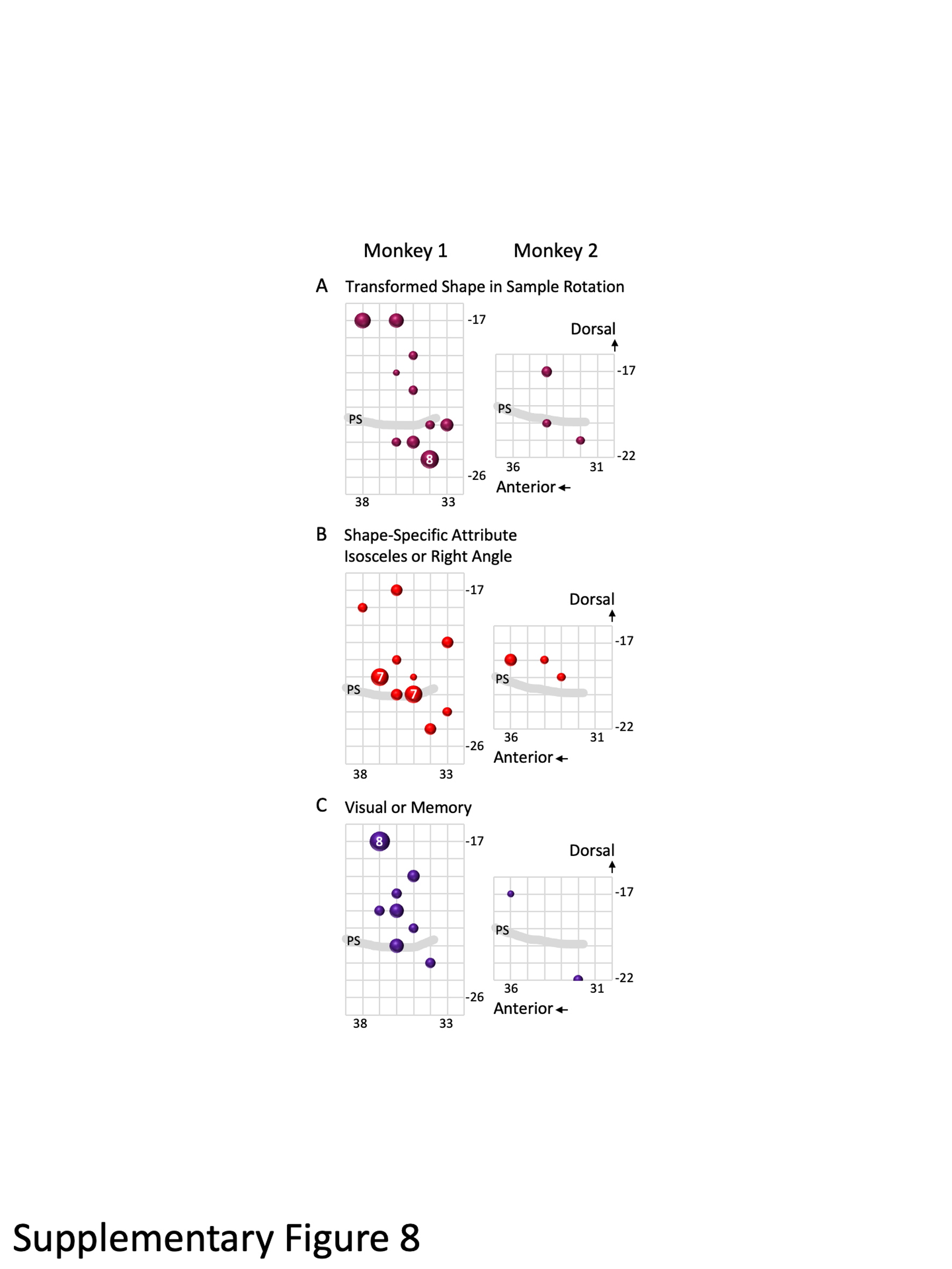

Supplement: Supplementary Figure 8 — Spatial distributions of STFM domains for Transformed Shape in Sample Rotation (A), Shape-Specific Attributes Isosceles or Right Rectangle (B), and Visual or Memory (C). The scale of the balls in (A,B) of shape-related variables is proportional to that in Figure 5F, and that in rule-related (C) is proportional to that in Figure 5G. [file Image_8.TIFF]
